# Interaction with Tsg101 Is Necessary for the Efficient Transport and Release of Nucleocapsids in Marburg Virus-Infected Cells

**DOI:** 10.1371/journal.ppat.1004463

**Published:** 2014-10-16

**Authors:** Olga Dolnik, Larissa Kolesnikova, Sonja Welsch, Thomas Strecker, Gordian Schudt, Stephan Becker

**Affiliations:** 1 Institut für Virologie, Philipps Universität Marburg, Marburg, Germany; 2 EMBL Structural and Computational Biology Unit, Heidelberg, Germany; 3 DZIF, Deutsches Zentrum für Infektionsforschung, Marburg, Germany; Mount Sinai School of Medicine, United States of America

## Abstract

Endosomal sorting complex required for transport (ESCRT) machinery supports the efficient budding of Marburg virus (MARV) and many other enveloped viruses. Interaction between components of the ESCRT machinery and viral proteins is predominantly mediated by short tetrapeptide motifs, known as late domains. MARV contains late domain motifs in the matrix protein VP40 and in the genome-encapsidating nucleoprotein (NP). The PSAP late domain motif of NP recruits the ESCRT-I protein tumor susceptibility gene 101 (Tsg101). Here, we generated a recombinant MARV encoding NP with a mutated PSAP late domain (rMARV_PSAPmut_). rMARV_PSAPmut_ was attenuated by up to one log compared with recombinant wild-type MARV (rMARV_wt_), formed smaller plaques and exhibited delayed virus release. Nucleocapsids in rMARV_PSAPmut_-infected cells were more densely packed inside viral inclusions and more abundant in the cytoplasm than in rMARV_wt_-infected cells. A similar phenotype was detected when MARV-infected cells were depleted of Tsg101. Live-cell imaging analyses revealed that Tsg101 accumulated in inclusions of rMARV_wt_-infected cells and was co-transported together with nucleocapsids. In contrast, rMARV_PSAPmut_ nucleocapsids did not display co-localization with Tsg101, had significantly shorter transport trajectories, and migration close to the plasma membrane was severely impaired, resulting in reduced recruitment into filopodia, the major budding sites of MARV. We further show that the Tsg101 interacting protein IQGAP1, an actin cytoskeleton regulator, was recruited into inclusions and to individual nucleocapsids together with Tsg101. Moreover, IQGAP1 was detected in a contrail-like structure at the rear end of migrating nucleocapsids. Down regulation of IQGAP1 impaired release of MARV. These results indicate that the PSAP motif in NP, which enables binding to Tsg101, is important for the efficient actin-dependent transport of nucleocapsids to the sites of budding. Thus, the interaction between NP and Tsg101 supports several steps of MARV assembly before virus fission.

## Author Summary

Marburg virus (MARV) is endemic in central Africa and causes hemorrhagic fever in humans and non-human primates, with high lethality. Presumably, the disease severity primarily depends on the response of host-cell factors interacting with viral proteins. We generated a recombinant MARV encoding an NP with a mutated PSAP late domain motif, which has previously been shown to mediate interaction with the cellular ESCRT protein Tsg101. We found that the PSAP-mediated interaction with Tsg101 was important at several steps of MARV assembly before viral fission. First, the egress of mature rMARV_PSAPmut_ nucleocapsids from viral inclusions was inhibited. Second, actin-driven transport of rMARV_PSAPmut_ nucleocapsids was impaired, displaying significantly shortened trajectories and reduced movement in the cell periphery. Third, rMARV_PSAPmut_ nucleocapsids accumulated in cell periphery, and the number of filopodia-associated nucleocapsids decreased, indicating that rMARV_PSAPmut_ nucleocapsids were defective to enter filopodia, the major budding sites of MARV. These defects resulted in the attenuated growth of rMARV_PSAPmut_. Interestingly, IQGAP1, an actin cytoskeleton regulator which interacts with Tsg101, was also recruited to nucleocapsids in dependence of the PSAP late domain. Thus, the interaction of NP with Tsg101 not only impacts viral budding at the plasma membrane but also nucleocapsid transport through the cytoplasm.

## Introduction

Tsg101 is a component of the endosomal sorting complex required for transport (ESCRT) machinery that mediates biogenesis of multi-vesicular bodies, specifically the formation and scission of the intraluminal vesicles, and is thus essential for the degradation and recycling of plasma membrane resident receptors [1]. In addition, ESCRT has been shown to function in the late steps of cytokinesis and to support the efficient budding of several enveloped viruses at the plasma membrane [2], [3]. For many viruses, Tsg101 serves as the central player for mediating the interaction between viral matrix proteins and the ESCRT machinery [4], [5]. This interaction is mediated by a tetrapeptide motif, PT/SAP, referred to as late domain because its mutation impairs viral release at a late stage of budding [4], [6]. The late domain phenotype is best characterized in retroviral infections in which viral particles are arrested in the budding process upon mutation of the PTAP motif in Gag and remain connected to the infected cell by only a thin membrane stalk [2]. Recently, Tsg101 has been reported to interact with Rab11 interacting effectors, the class II Rab11-FIPs, suggesting a functional link between transport pathways and the ESCRT machinery [7]. Additionally, Tsg101 interacts with regulators of cytoskeleton dynamics, such as IQGAP and ROCK1 [8], and is essential for translocating the tyrosine kinase Src to cellular protrusions [9]. Together, these results indicate a role for Tsg101 in intracellular transport processes. Tsg101 expression and functions are highly regulated by an intrinsic autoregulatory mechanism and by ubiquitination involving several distinct ubiquitin ligases [10]–[14]. A cargo-dependent degradation of Tsg101 as a feedback mechanism for modulating endosomal sorting has also been described [15].

Marburg virus (MARV), a filovirus, causes severe hemorrhagic fever in humans and non-human primates, with mortality rates of up to 90% [16]. Despite advances in experimental vaccine approaches, no vaccine or antiviral treatment against filoviral hemorrhagic fever are available for human use [17]. The filamentous MARV particles, which are approximately 900 nm in length and 90 nm in diameter, are composed of seven proteins. Five viral proteins are associated with the nucleocapsid. The nucleoprotein (NP) encapsidates the non-segmented negative-strand RNA genome and forms long helical nucleocapsids together with the viral proteins VP35, L, VP30 and VP24 [18], [19]. The nucleocapsid is surrounded by a layer of the matrix protein VP40, which is associated with the viral envelope, where homotrimers of the surface glycoprotein (GP) are inserted [18], [20].

Virogenesis starts with the appearance of inclusions in the cytosol, which represent accumulations of MARV nucleocapsids [21], [22]. For Ebola virus, the other member of the *Filoviridae* family, the inclusions have recently been shown to represent sites of viral replication [23]. Ultrastructural analysis has revealed that the inclusions contain nucleocapsids of variable electron density. Nucleocapsids of higher electron density are transported to the plasma membrane, where they become associated with VP40 and enveloped by budding through the GP-enriched plasma membrane [22], [24]–[26]. Viral budding primarily occurs at the sides or tips of filopodia [22], [27], [28]. The intracellular transport of nucleocapsids is driven by the polymerization of actin [26].

The efficient release of VLPs induced by VP40, the key player in MARV assembly and budding, requires the support of the ESCRT machinery that is recruited to VP40 by the late domain motif PPPY [29], [30]. Interestingly, the co-expression of NP strongly enhanced the release of VP40-induced VLPs [29], [30]. Our previous data showed that a PSAP late domain in the C-terminus of NP was responsible for this enhancing effect by recruiting Tsg101 to the plasma membrane [31]. Here, we investigated these results in the context of MARV infection using reverse genetics.

Our study revealed that recombinant MARV with a mutated PSAP late domain motif in NP (rMARV_PSAPmut_) could not recruit Tsg101 to the nucleocapsids, was attenuated in growth and impaired in cell-to-cell spread. By using several experimental approaches, we found that rMARV_PSAPmut_ displayed a novel late domain phenotype with an altered morphology of the viral inclusions and an accumulation of nucleocapsids in the cytoplasm, at the plasma membrane and in the process of envelopment. In addition, nucleocapsids in rMARV_PSAPmut_–infected cells had an altered pattern of movement most likely due to reduced recruitment of IQGAP1, a regulator of actin dynamics. Together, these data indicate the involvement of the NP late domain at several pre-fission steps during MARV assembly.

## Results

### Tsg101 knockdown in MARV-infected cells results in reduced particle release

**Figure 1:**
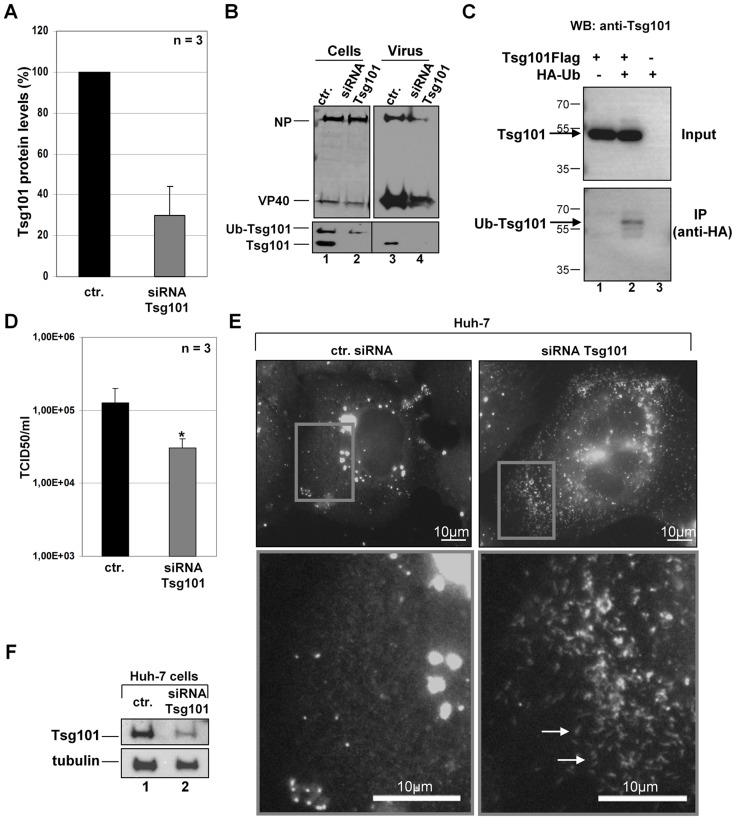
Tsg101 knockdown in MARV-infected cells results in reduced particle release. MARV-infected Huh-7 cells (MOI of 1) were transfected twice with Tsg101-specific siRNA or control siRNA (1 h and 18 h p.i.). Cells and virus particles were harvested at 48 h p.i. (**A**) Quantification of Tsg101 protein level was performed by Western Blot in cells transfected with Tsg101-specific siRNA and control siRNA. Tsg101 levels in cells transfected with control siRNA (ctr.) were set to 100%. (**B**) Lysates and supernatants of MARV infected cells transfected with Tsg101-specific or control siRNA were subjected to Western Blot and analyzed for the presence of viral proteins NP and VP40 and Tsg101. (**C**) The 65 kDa form of Tsg101 is ubiquitinated. HEK293 cells were transfected with Tsg101-Flag and HA-Ubiquitin expression plasmids. At 48 h p.tr., cell lysates were subjected to immunoprecipitation with anti-HA-agarose. Cell lysates and precipitates were separated by SDS-PAGE and analyzed by Western Blot using HA- and Tsg101-specific antibodies. The position of the ubiquitinated Tsg101 (Ub-Tsg101) band is indicated by an arrow between 55 and 70 kDa. (**D**) Virus titers in the supernatants of MARV infected cells transfected with Tsg101-specific or control siRNA were determined by TCID_50_ titration, p-value (_*_, P≤0.05). (**E**) **P**henotype of Tsg101 knockdown in MARV-infected cells. Huh-7 cells were infected with MARV and treated with Tsg101 specific or control siRNA and subjected to immunofluorescence analysis using a guinea pig anti-NP and secondary goat anti-guinea pig FITC-conjugated antibody. Grey boxes indicate marginal region of cells. Lower panels show higher magnification of boxed area, arrows indicate accumulation of nucleocapsids upon Tsg101 knockdown. Bars, 10 µm. (**F**) Western blot analysis of Tsg101 knockdown. Cells transfected with Tsg101-specific or control siRNA were analyzed by Western Blot using Tsg101- and tubulin-specific antibodies.

Tsg101 interacts with the MARV NP through a C-terminal PSAP late domain. The mutation of this domain impairs binding of Tsg101 and simultaneously abolishes the positive impact of NP on the release of VLPs induced by the MARV matrix protein VP40 [31]. To further analyze the function of Tsg101 during MARV infection, we down-regulated Tsg101 expression in MARV-infected cells using small interfering RNA (siRNA) technology. Tsg101-specific siRNA reduced the levels of Tsg101 expression to 30% compared with control siRNA (Fig. 1A). Western blot analysis of the cell lysates detected two prominent forms of Tsg101 at 46 and 65 kDa, respectively (Fig. 1B, lane 1). Transfection of Tsg101-specific siRNA reduced the levels of both forms of Tsg101, and Tsg101 incorporation into viral particles was reduced to undetectable levels (Fig. 1B, lanes 3 and 4). Because Tsg101 (46 kDa) can be multi-monoubiquitinated, it was presumed that the 65-kDa form represented ubiquitinated Tsg101 [10], [12]–[14]. To confirm this presumption, Flag-tagged Tsg101 and HA-tagged ubiquitin (HA-Ub) were co-expressed in HEK293 cells, and the cell lysates were used for immunoprecipitation with anti-HA agarose. Western blot analysis of the cell lysates using an anti-Tsg101 antibody mainly revealed the expected 46-kDa form of Tsg101 (Fig. 1C, lanes 1 and 2, upper panel). Immunoprecipitation of HA-tagged ubiquitinated cellular proteins and staining of the precipitates with anti-Tsg101 antibody revealed Tsg101-specific proteins with a major signal at approximately 65 kDa, corresponding to multi-ubiquitinated Tsg101 (Fig. 1C, lane 2, lower panel). Although the intracellular amounts of viral proteins remained unaffected by Tsg101 knockdown (Fig. 1B, lane 2), the release of viral proteins was reduced to 32%±18.6% for NP and 44%±10.4% for VP40 (n = 3; Fig. 1B, lane 4). Corresponding with this impaired release of viral proteins, the viral titers in the supernatants of Tsg101-depleted cells were 3- to 4-fold lower than in the control cells (Fig. 1D). Additionally, Tsg101 depletion resulted in the accumulation of nucleocapsids in the cytoplasm. Although 15% of the infected and Tsg101-depleted cells showed intracytoplasmic accumulation of nucleocapsids, only 1.5% of the infected cells treated with the control siRNA showed a similar phenotype (Fig. 1E–F, white arrows, and lower panel right). Together, these results support the hypothesis that Tsg101 is needed for the efficient release of MARV nucleocapsids, and correspondingly, a lack of Tsg101 leads to the intracellular accumulation of nucleocapsids.

### Recovery and characterization of recombinant MARV_PSAPmut_

**Figure 2:**
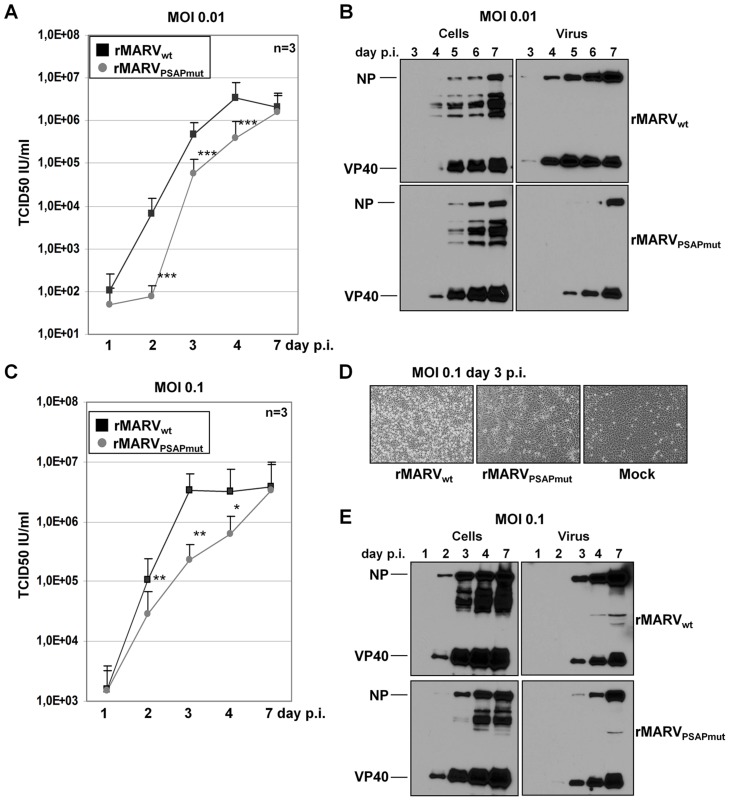
rMARV_PSAPmut_ exhibits delayed growth kinetics. Vero E6 cells were inoculated with either rMARV_PSAPmut_ or rMARV_wt_. Supernatants and cell lysates were collected at indicated time points p.i. and viral titres determined by TCID_50_ assay or subjected to Western blot analysis. (**A**) Growth kinetics of rMARV_PSAPmut_ (grey circle) or rMARV_wt_ (black square) at MOI of 0.01. (**B**) Western Blot analysis of viral protein levels during an infection at MOI of 0.01. Cell lysates and culture supernatants were collected at indicated time points and were analyzed by SDS-PAGE and Western Blotting using NP- and VP40-specific antibodies. (**C**) Growth kinetics of rMARV_PSAPmut_ (grey circle) or rMARV_wt_ (black square) at MOI of 0.1. (**D**) rMARV_PSAPmut_- or rMARV_wt_– or mock-infected cells were analyzed for CPE formation during infection at MOI of 0.1 at 3 days p.i. (**E**) Western Blot analysis of viral protein levels during an infection at MOI of 0.1. Cell lysates and culture supernatants were collected at indicated time points and were analyzed as described in Fig. 1B. P-values are indicated (_*_, P≤0.05; _**_, P≤0.001; _***_, P≤0.0001).

**Figure 3:**
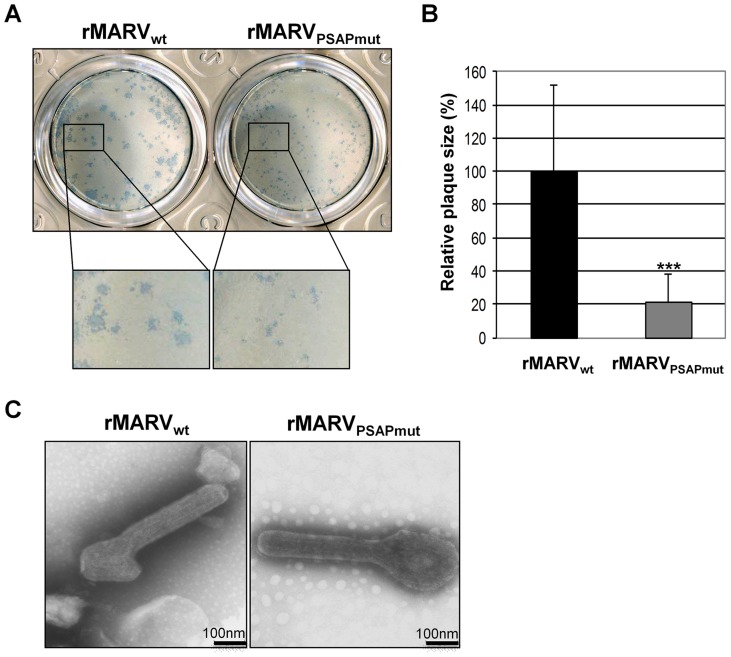
rMARV_PSAPmut_ shows reduced plaque size but unaltered particle morphology. (**A**) Vero E6 cells were infected with rMARV_PSAPmut_ and rMARV_wt_ and plaque formation monitored at 4 d p.i. by immunostaining. (**B**) Statistical analysis of the plaque size (n = 37). rMARV_wt_ plaque size was set to 100%, p-value (_***_, P≤0.0001). (**C**) Morphology of viral particles. rMARV_PSAPmut_ and rMARV_wt_ viral particles were fixed, negatively stained with 2% phosphotungstic acid and analyzed by electron microscopy.

Because Tsg101 is a multifunctional protein that is involved in several cellular pathways, its down-regulation may have multiple effects on viral replication that are not directly related to its interaction with MARV proteins [32]. We therefore specifically inhibited the interaction of Tsg101 with NP by mutating the C-terminal PSAP motif of NP in the MARV genome using reverse genetics [33]. Our previous studies revealed that this mutation significantly inhibited the interaction between NP and Tsg101 [31]. The mutated virus (rMARV_PSAPmut_) was rescued, and its phenotype was analyzed (Fig. 2). The rMARV_PSAPmut_ growth kinetics at a low MOI (0.01) were reduced, and the measured TCID_50_ titers were between one and two logs lower than for rMARV_wt_ (Fig. 2A). These differences were reflected in the amount of viral protein in the supernatant of infected cells. At all tested time-points post-infection (p.i.), the rMARV_PSAPmut_-infected cells released less VP40 and NP than the rMARV_wt_-infected cells, whereas the cellular amounts of viral proteins remained similar, indicating that rMARV_PSAPmut_ and rMARV_wt_ had similar RNA synthesis and translation rates (Fig. 2B, left). These results were in line with previous results showing that the PSAP mutation does not affect NP activity in transcribing/replicating a MARV-specific minigenome [31]. A delay in the release of rMARV_PSAPmut_ particles was also observed at a higher MOI (0.1). At three days p.i., cytopathic effects were less pronounced in the cells infected with rMARV_PSAPmut_ than with rMARV_wt_ (Fig. 2C–D). We then analyzed the supernatants of rMARV_PSAPmut_-infected cells and found that although the release of VP40 was comparable with rMARV_wt_, the NP levels were severely reduced (Fig. 2E). This result suggests that the budding activity of VP40 is most likely not affected by mutation of the PSAP motif in NP. In contrast, NP incorporation into particles is reduced, resulting in the release of less infectious particles (Fig. 2E). Together, these results suggested a defect in the release of infectious rMARV_PSAPmut_ particles. This idea was supported by the analysis of plaque sizes formed by the different viruses. Plaques produced by rMARV_PSAPmut_ were 5-fold smaller than those produced by rMARV_wt_ (Fig. 3A–B). A comparison of the released viral particles by electron microscopy showed no morphological difference between rMARV_PSAPmut_ and rMARV_wt_ virions (Fig. 3C).

### rMARV_PSAPmut_ particles incorporate less Tsg101 than rMARV_wt_ particles

**Figure 4:**
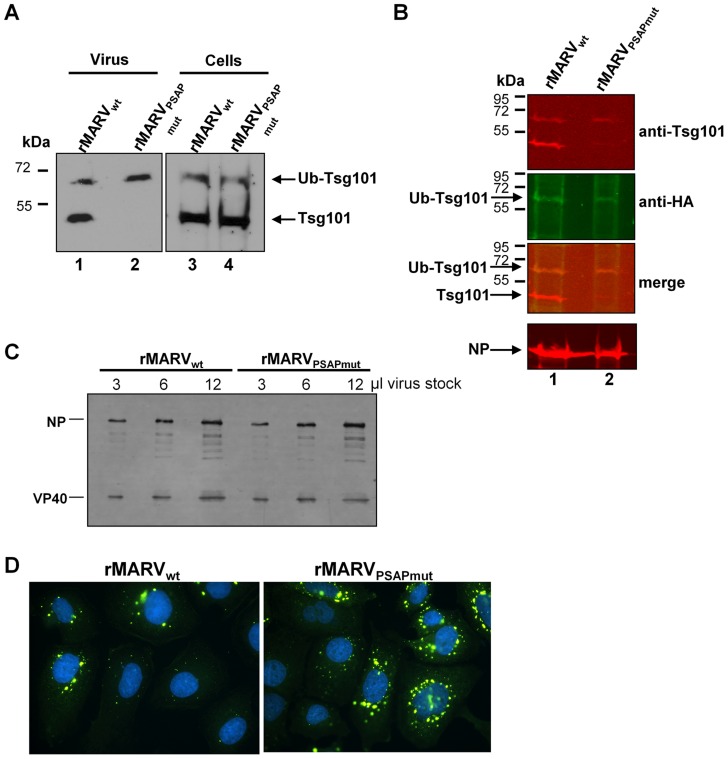
rMARV_PSAPmut_ particles incorporate less Tsg101 and display similar infectivity. (**A**) Tsg101 incorporation into MARV particles. Vero E6 cells were infected with rMARV_wt_ or rMARV_PSAPmut_ and virus particles released into the supernatant were pelleted through a 20% sucrose cushion at 48 h p.i. Virus pellets and cell lysates were subjected to SDS-PAGE and Western Blot analysis using Tsg101-specific antibody. (**B**) Detection of ubiquitinated form of Tsg101 in viral particles. Vero E6 cells were infected with rMARV_wt_ or rMARV_PSAPmut_ and subsequently transfected with HA-Ub expression plasmid. Virus particles were pelleted from the supernatants and analyzed by SDS-PAGE and Western Blot analysis using anti-Tsg101 and anti-HA specific primary antibodies and secondary antibodies for detection with the Odyssey imaging system (see merge image). (**C, D**) Comparison of virus infectivity. (**C**) Equal amounts of TCID_50_ units of rMARV_PSAPmut_ and rMARV_wt_ stock viruses were pelleted through 20% sucrose cushion, separated by SDS-PAGE and analyzed by Western Blot using NP- and VP40-specific antibodies. (**D**) Huh-7 cells grown on glass cover slips were inoculated with rMARV_PSAPmut_ and rMARV_wt_ normalized to nucleoprotein amount, fixed at 17 h p.i. and stained with DAPI and NP-specific antibody for detection of infected cells by immunofluorescence assay.

Our previous study revealed that Tsg101 was incorporated into purified MARV particles [31]. To verify that the mutation of the PSAP late domain in NP specifically impaired the interaction with Tsg101, we analyzed whether rMARV_PSAPmut_ particles remained able to incorporate Tsg101. Lysates from infected cells and released virus particles were subjected to Western blot analyses. In the lysates from rMARV_PSAPmut_- and rMARV_wt_-infected cells, both the non-ubiquitinated (46 kDa) and multi-ubiquitinated (65 kDa) forms of Tsg101 were detected. Immunoprecipitation of infected cells transfected with Tsg101-Flag and HA-Ub confirmed that the 65-kDa form of Tsg101 represents multi-ubiquitinated Tsg101 (Fig. S1, lane 3). Both forms of Tsg101 were incorporated into rMARV_wt_ particles (Fig. 4A, lane 1). In contrast, rMARV_PSAPmut_ particles mainly incorporated the ubiquitinated form of Tsg101 (Fig. 4A, lane 2). To confirm that ubiquitinated Tsg101 is indeed incorporated into virus particles, rMARV_wt_- and rMARV_PSAPmut_-infected cells were transfected with HA-Ub, and the released virus particles were purified from culture supernatants and analyzed for the presence of Tsg101, HA-Ub and NP. Reactivity with an HA-specific antibody indicated that the 65-kDa Tsg101 form was ubiquitinated (Fig. 4B, anti-HA). Ubiquitinated Tsg101 was incorporated at low levels in both rMARV_wt_ and rMARV_PSAPmut_ particles. In contrast, non-ubiquitinated Tsg101 was the prominent form in rMARV_wt_ particles and nearly undetectable in rMARV_PSAPmut_ particles (Fig. 4B, anti-Tsg101). In conclusion, these data indicate that the PSAP late domain of MARV NP specifically recruits non-ubiquitinated Tsg101 into viral particles.

We then investigated whether the reduced amount of Tsg101 in rMARV_PSAPmut_ particles altered virus infectivity. rMARV_PSAPmut_ and rMARV_wt_ particles, each at a concentration of 6×10^2^ TCID_50_/µl, were subjected to Western blot analysis. Fig. 4C shows that the equal infectious doses of the two viruses corresponded with the same amount of viral proteins, indicating that the infectivity-to-particle ratio between the two viruses was similar. This was confirmed by infection of Huh-7 cells with rMARV_wt_ and rMARV_PSAPmut_ suspensions, which were normalized to equal amounts of NP (Fig. 4D). Infected cells were fixed at 17 h p.i., which corresponds to the duration of one viral replication cycle, and the cells were subsequently immuno-stained with an anti-MARV polyclonal antibody. The number of infected cells was higher for rMARV_PSAPmut_ compared with rMARV_wt_ (1.7±0.4-fold more rMARV_PSAPmut_-infected cells, n = 4), indicating that the rMARV_PSAPmut_ was not less infectious than the wild-type MARV (Fig. 4D). In summary, these data show that the presence of Tsg101 in viral particles does not influence viral infectivity, suggesting that the attenuation of rMARV_PSAPmut_ is likely caused by reduced release of viral particles.

### rMARV_PSAPmut_ nucleocapsids accumulate in inclusion bodies and in the cytoplasm

**Figure 5:**
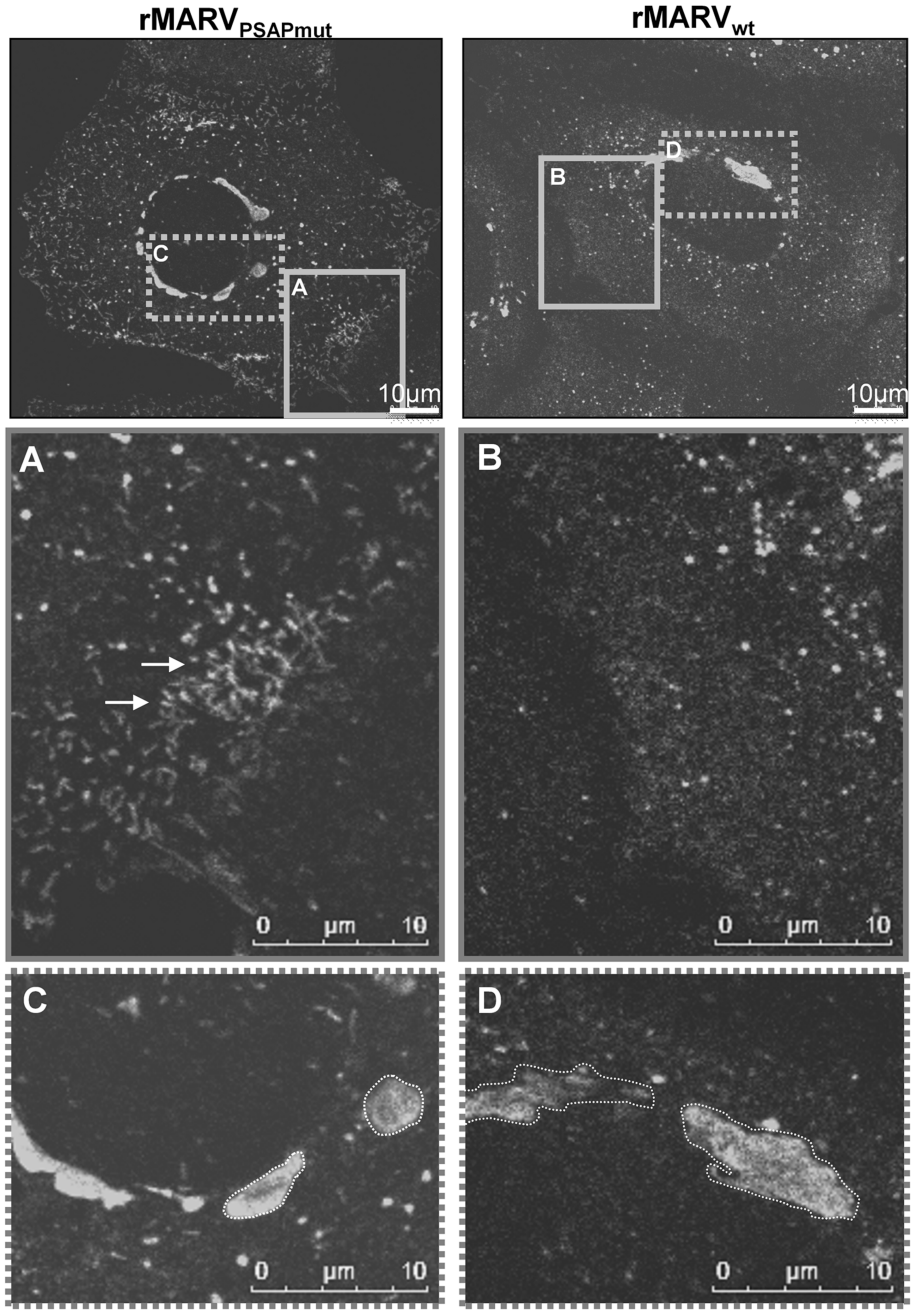
Infection with MARV_PSAPmut_ results in compact inclusion bodies and accumulation of nucleocapsids in the periphery of cells. Huh-7 cells were infected with rMARV_wt_ or rMARV_PSAPmut_, fixed at 24 h p.i. and subjected to immunofluorescence staining using NP-specific antibodies. Samples were then analyzed by confocal laser scanning microscopy. Left panels: rMARV_wt_ infection. Right panels: rMARV_PSAPmut_ infection. Grey boxes in the upper pictures indicate different regions of the same cell that are shown in higher magnification below. (**A**) and (**B**) periphery of cells. (**C**) and (**D**) inclusion bodies. Arrows indicate nucleocapsids.

We next analyzed which step in the transport and release of nucleocapsids was impaired by the missing interaction with Tsg101. Because depletion of Tsg101 led to an accumulation of cytoplasmic MARV nucleocapsids (Fig. 1E), we tested whether nucleocapsids in rMARV_PSAPmut_-infected cells exhibited a similar phenotype (Fig. 5). Indeed, indirect immunofluorescence analysis revealed an accumulation of elongated filamentous structures, mostly in the periphery of rMARV_PSAPmut_-infected cells (Fig. 5A, white arrows). Based on their NP content and dimensions, these structures have recently been identified as nucleocapsids [26]. Cells infected with rMARV_wt_ did not exhibit this accumulation of nucleocapsids (Fig. 5B). In addition, although inclusions in rMARV_wt_-infected cells were pleomorphic (Fig. 5D), inclusions in rMARV_PSAPmut_-infected cells were more round and compact and frequently exhibited a bright NP signal at their rim (Fig. 5C).

**Figure 6:**
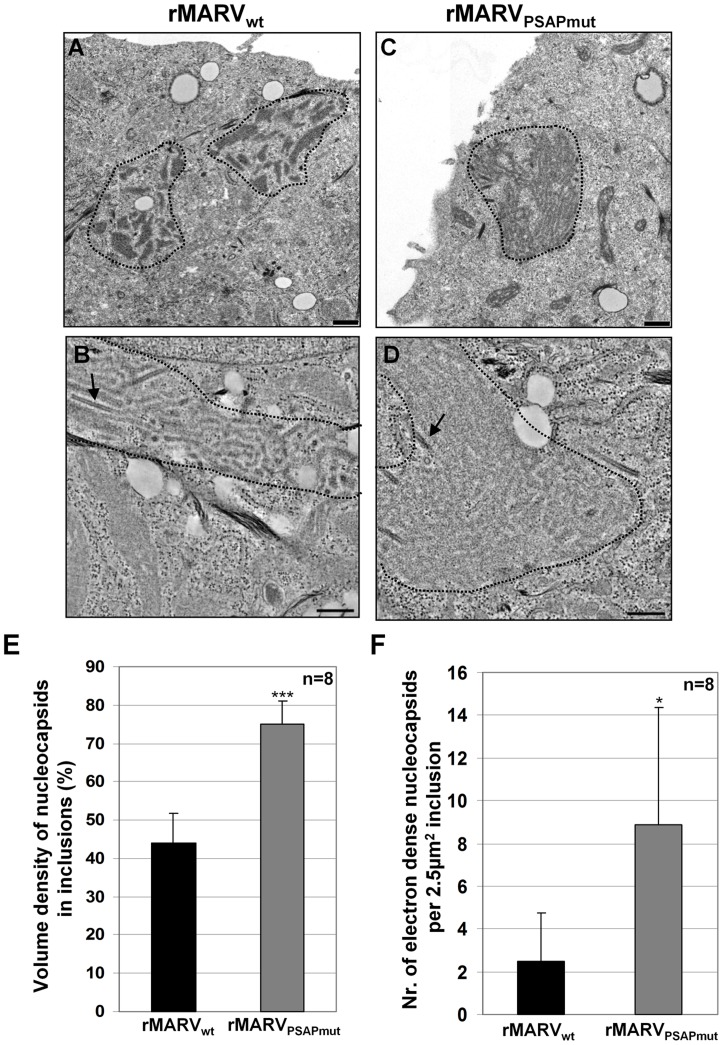
Inclusions in rMARV_PSAPmut_–infected cells are more densely packed with nucleocapsids than inclusions in rMARV_wt_–infected cells. Huh-7 cells were infected with rMARV_wt_ or rMARV_PSAPmut_. At 28 h p.i., cells were processed in two ways (i) fixed, scraped, pelleted and then embedded in Epoxy resin (A and C); or (ii) fixed and embedded in Epoxy resin on Thermanox slides (B and D). Ultrathin sections were stained with uranyl acetate and subjected to electron microscopy. (**A–B**) rMARV_wt_–infected cells, (**C–D**) rMARV_PSAPmut_–infected cells. Bars, 500 nm. (**E**) Morphometric analysis of inclusions. Volume density of nucleocapsids inside inclusions is shown, p-value (***, p≤0.0001). (**F**) Amount of electron dense (mature) nucleocapsids inside inclusions (see black arrows Fig. 6B und D) determined per 2.5 µm^2^ of inclusion at electron micrographs, p-value (*, p≤0.05).

Using electron microscopy, we further analyzed how mutation of the PSAP motif affected the morphology of viral inclusions. These analyses confirmed that inclusions in rMARV_wt_-infected cells primarily appeared as a disperse pleomorphic viroplasm in which nucleocapsids were packed with variable density, similar to the MARV inclusions described previously (Fig. 6A–B) [21], [22], [34]. In contrast, the majority of viral inclusions in rMARV_PSAPmut_-infected cells had a compact and spherical appearance, and they always contained densely packed nucleocapsids (Fig. 6C–D). Using the stereological morphometry of electron micrographs, we quantitatively determined the volume density of nucleocapsids. Packing of nucleocapsids was 1.7-fold higher in inclusions from rMARV_PSAPmut_-infected cells than in those from rMARV_wt_-infected cells (75%±6% and 44%±8%, respectively, Fig. 6E). Electron-dense nucleocapsids were detected 3.3-fold more frequently in inclusions from rMARV_PSAPmut_-infected cells compared with rMARV_wt_-infected cells (8.6±5 and 2.6±2 per 2.5 µm^2^, respectively, Fig. 6F). These results confirmed the immunofluorescence analyses and indicated that the missing interaction between Tsg101 and NP modifies the morphodynamics of viral inclusions.

**Figure 7:**
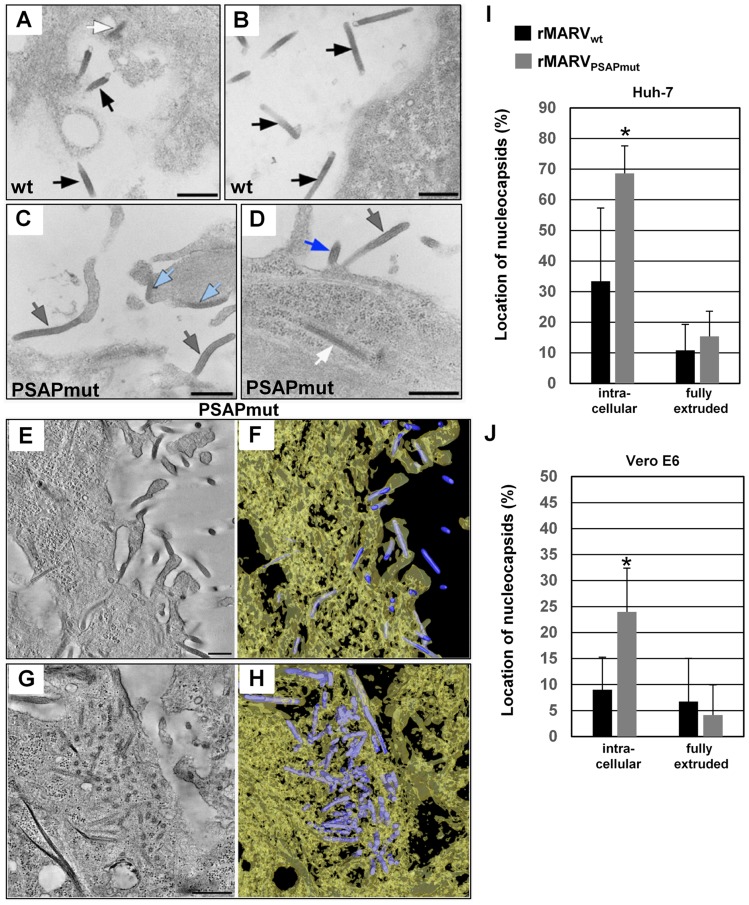
rMARV_PSAPmut_-infected cells contain more nucleocapsids in the cytoplasm and at early steps of budding than rMARV_wt_-infected cells. Huh-7 and Vero E6 cells were infected with rMARV_PSAPmut_- or rMARV_wt_, fixed at 26 h p.i and embedded in epoxy resin. Cells were analyzed by thin section transmission electron microscopy (**A–D**) or electron tomography (**E–J**). (**A–B**) rMARV_wt_-infected Huh-7 cell displaying free virions (black arrows) and a nucleocapsid in the cytoplasm near the plasma membrane (white arrow in A). (**C–D**) rMARV_PSAPmut_-infected Huh-7 cells displaying fully protruded (grey arrows) or partially protruded (blue arrow in D) virus buds, and nucleocapsids bound to the plasma membrane (light blue arrows in C) or in the cytoplasm near the plasma membrane (white arrow in D). (**E**) 10 nm digital z-slice of an electron tomogram showing several nucleocapsids in the process of budding or in fully protruded virus buds in the periphery of rMARV_PSAPmut_-infected Huh-7 cell. (**G**) 9 nm digital z-slice of an electron tomogram showing accumulated nucleocapsids in the cytoplasm of rMARV_PSAPmut_-infected Huh-7 cell. (**F, H**) 3D surface representations of nucleocapsids (blue) and cytoplasm (yellow, semi-transparent) in the full tomograms for which z-slices are shown in Fig. 7E and 7G, respectively. Bars, 500 nm. (**I–J**) Quantification of the nucleocapsid distribution in tomograms from 300 nm thick sections of rMARV_wt_- or rMARV_PSAPmut_-infected Huh-7 or Vero E6 cells. Intracellular nucleocapsids (including cytoplasmic and those bound to plasma membrane, or partially extruded nucleocapsids) and fully extruded nucleocapsids were counted in a set (5 or more) of representative tomograms (p-value, *P≤0.05).

We next assessed the localization of nucleocapsids outside viral inclusions in thin sections of infected cells. In rMARV_wt_-infected cells, mature nucleocapsids were mainly detected in released virions (Fig. 7A–B). In contrast, in rMARV_PSAPmut_ infected cells nucleocapsids were frequently detected in the cytosol, close to the cell surface and in protruding buds (Fig. 7C–D). Because the association of cytoplasmic nucleocapsids and budding viruses with membranes can be assessed unequivocally for only full-length nucleocapsids, we quantified the nucleocapsid distributions in tomograms reconstructed from thick sections of rMARV_wt_- and rMARV_PSAPmut_-infected cells (Fig. 7E–J). Intracellular nucleocapsids (outside the inclusions) were detected at three different locations: (i) cytoplasmic, without any connection to the plasma membrane (white arrow in 7A and 7D); (ii) plasma-membrane-bound nucleocapsids attached on one side to the plasma membrane (light-blue arrows in 7C); (iii) protruding buds with partially enveloped nucleocapsids (blue arrow in 7D). We differentiated those intracellular nucleocapsids from nucleocapsids that were completely enveloped but still had a thin connection to the plasma membrane (grey arrows in 7C–D). The latter phenotype had been referred to as late domain phenotype in previous publications [2]. Finally, we detected nucleocapsids in the extracellular space corresponding to free virus (i.e., nucleocapsid inside released viral particles; black arrows in 7A and 7B). The relative amount of intracellular nucleocapsids was significantly higher in cells infected with rMARV_PSAPmut_ than in cells infected with rMARV_wt_ (Fig. 7I–J). Interestingly, the percentage of fully protruded buds was not different for both viruses, which argued against a classical fission defect of rMARV_PSAPmut_ [2]. Together, the ultrastructural data are in line with the immunofluorescence analyses (Fig. 5A–B) indicating that the missing interaction with Tsg101 retards nucleocapsids in the cytoplasm and/or slows down envelopment of the nucleocapsids at the plasma membrane rather than influencing the final fission step.

### The recruitment of nucleocapsids into filopodia is reduced in MARV_PSAPmut_-infected cells

**Figure 8:**
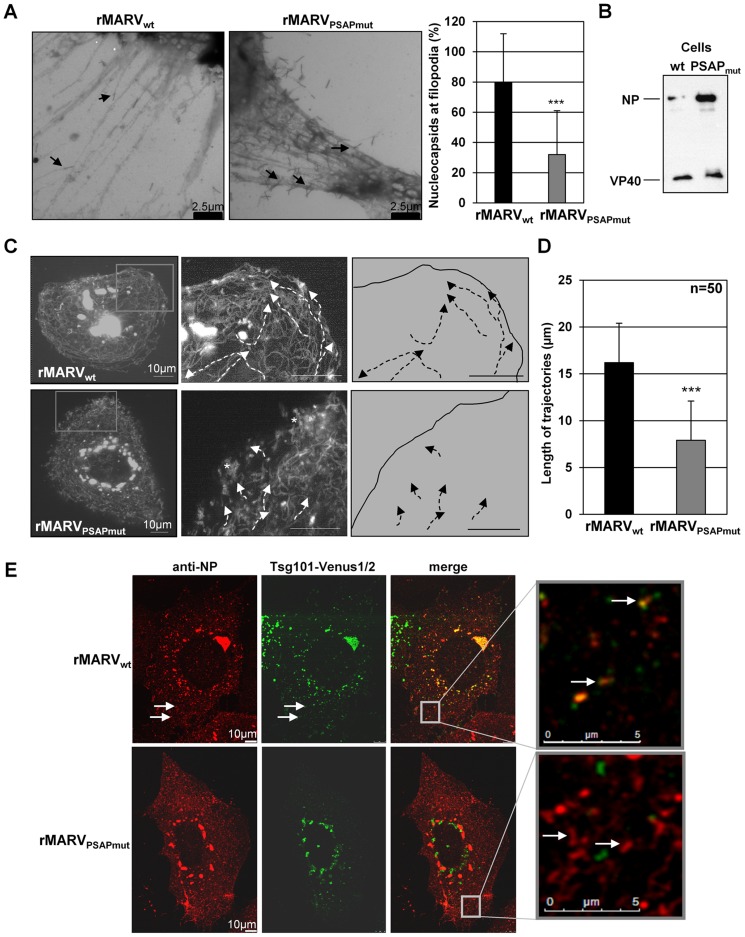
Budding from filopodia is reduced in rMARV_PSAPmut_-infected cells. (**A**) Huh-7 cells were infected with rMARV_wt_ or rMARV_PSAPmut_ at an MOI of 1, and fixed at 19–23 h p.i. The whole mounted cells were analyzed by electron microscopy on grids after negative staining. Graphics shows the percentage of nucleocapsids in the process of budding from the filopodia (20 micrographs were analyzed for each virus), p-value (*** P≤0.0001). (**B**) Western Blot analysis of viral proteins in cell lysates. Huh-7 cells were infected as indicated in (A), harvested at 19 h p.i., and analyzed by using NP- and VP40-specific antibodies. (**C**) Live cell imaging of Huh-7 cells were infected with rMARV_wt_- or rMARV_PSAPmut_. At 28 h (rMARV_PSAPmut_) and 43 h p.i. (rMARV_wt_), a series of 600 pictures was taken every second for a period of 10 min. Maximal projection of the picture series is displayed. Boxes in the left panels indicate areas that are shown at higher magnification in the middle panels. Trajectories of individual nucleocapsids are highlighted with white dashed lines. White asterisks indicate regions with several static rMARV_PSAPmut_ nucleocapsids. Right panels show the trajectories of nucleocapsids in rMARV_wt_- or rMARV_PSAPmut_-infected cells. Bars, 10 µm. (**D**) Length of nucleocapsid trajectories. The length of nucleocapsid trajectories was measured in rMARV_wt_- or rMARV_PSAPmut_-infected cells using the Leica LAS AF software, p-value (***, p≤0.0001). (**E**) Co-localization of Tsg101-Venus1/2 with MARV inclusions and nucleocapsids. Huh-7 cells were infected either with rMARV_wt_ or rMARV_PSAPmut_ and transfected with Venus1-Tsg101 and Venus2-Tsg101 plasmids. Cells were fixed at 22 h p.i. and subjected to immunofluorescence analysis with a NP-specific antibody. The Tsg101-Venus1/2 signal is displayed in green and the NP signal in red. Merged pictures show the overlay. The grey boxes indicate marginal region of cells, which are shown at higher magnification in the right panels. Arrows indicate nucleocapsids (approximately 1 µm in length).

The release of MARV nucleocapsids occurs by budding at the side or tips of filopodia, which is difficult to detect in tomograms of semi-thick sections [18], [27], [28]. Localization of nucleocapsids in filopodia was therefore analyzed by electron microscopy of whole-mounted cells (Fig. 8). Using this method, most of the budding rMARV_wt_ nucleocapsids were found in association with filopodia (80%, Fig. 8A). In contrast, only 32% of budding nucleocapsids in rMARV_PSAPmut_-infected cells were detected in association with filopodia. The majority of nucleocapsids bud at the planar cell surface (68%). Western Blot analysis of virus infected cells confirmed prominent accumulation of NP in MARV_PSAPmut_-infected cells at 19 h p.i. (Fig. 8B).

### Tsg101 is involved in the transport of MARV nucleocapsids

We then analyzed the trajectories of migrating nucleocapsids by live-cell imaging. Cells were infected either with rMARV_PSAPmut_ or rMARV_wt_ and then transfected with a plasmid encoding the nucleocapsid-associated protein, VP30, fused to the green fluorescent protein, GFP (VP30-GFP). Fluorescent VP30-GFP was previously shown to be efficiently associated with nucleocapsids to allow monitoring of nucleocapsid transport in real time by live-cell imaging [26]. The transport speed of the rMARV_PSAPmut_ nucleocapsids was not significantly different from that of the rMARV_wt_ nucleocapsids [304±99 (n = 34) and 313±116 nm/s (n = 39) for rMARV_wt_ and rMARV_PSAPmut_, respectively]. However, the pattern of nucleocapsid transport was altered. rMARV_wt_ nucleocapsids displayed directed movement from the cell center to the cell margins and along the plasma membrane over long distances in line with previous observations [16.2±4.2 µm (n = 50), Fig. 8D] [26]. In contrast, the observed trajectories for nucleocapsids in rMARV_PSAPmut_-infected cells were considerably shorter [7.9±4.95 µm (n = 50)], and many nucleocapsids located beneath the plasma membrane exhibited an immobile phenotype (Fig. 8C, white asterisk middle panel, and movie S1).

To further analyze the role of Tsg101 in the transport of nucleocapsids, a protein complementation assay was used to visualize functional Tsg101 in living cells [35], [36]. Fusion proteins of Venus-1 and Tsg101 and Venus-2 and Tsg101 were generated (Venus1-Tsg101 and Venus2-Tsg101) that show immunofluorescence signals only upon interaction of the two Venus fragments which is enabled when the fusion partner Tsg101 interacts with itself. The two Tsg101-Venus constructs were co-expressed in cells infected with either rMARV_wt_ or rMARV_PSAPmut_ and the intracellular localization of fluorescent oligomerized Tsg101-Venus1/2 was analyzed by confocal laser scanning microscopy (CLSM). Tsg101-Venus1/2 was localized in the inclusion bodies of rMARV_wt_-infected cells, whereas rMARV_PSAPmut_ inclusions did not accumulate Tsg101-Venus1/2 (Fig. 8E). In addition, Tsg101-Venus1/2 was co-localized with individual rMARV_wt_ nucleocapsids outside the inclusions, which was not observed in rMARV_PSAPmut_-infected cells (Fig. 8E, white arrows). We then investigated the behavior of Tsg101-Venus1/2 using live-cell imaging of rMARV_VP30RFP_-infected cells. During the observation period, Tsg101-Venus1/2-positive granular structures were increasingly accumulated within the viral inclusions (Fig. S2A and movie S2). In addition, co-transport of Tsg101-Venus1/2 and nucleocapsids was observed (movie S3). The maximal projection of the acquired sequences showed an overlay of the nucleocapsid signal with the Tsg101-Venus1/2 signal (Fig. S2B). Collectively, these data demonstrated a co-transport of Tsg101 with nucleocapsids and suggest that PSAP-dependent recruitment of Tsg101 by NP improves the transport of MARV nucleocapsids.

**Figure 9:**
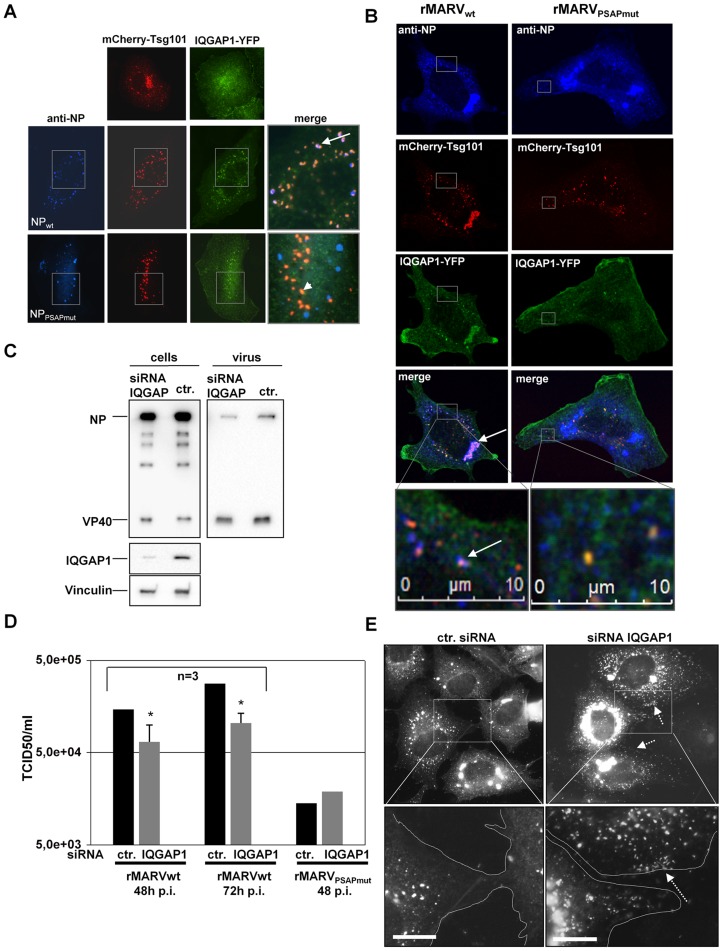
IQGAP1 is co-localized with inclusions and nucleocapsids and supports MARV release. (**A**) Co-localization of Tsg101 and IQGAP1 in NP inclusions. Huh-7 cells were co-transfected with plasmids encoding NP_wt_ or NP_PSAPmut_ and VP40, mCherry-Tsg101 and IQGAP1-YFP. Cells were fixed 24 h p.tr., stained with NP-specific antibody and subjected to immunofluorescence analysis. White arrow shows co-localization of mCherry-Tsg101 (red), IQGAP1-YFP (green) with NP inclusions (blue) in the merge picture. In NP_PSAPmut_ transfected cells only mCherry-Tsg101 and IQGAP1-YFP are co-localized (arrowhead). (**B**) Co-localization of Tsg101 and IQGAP1 in MARV infected cell. Huh-7 cells were infected with rMARV_wt_ or rMARV_PSAPmut_ and co-transfected with mCherry-Tsg101 and IQGAP1-YFP encoding plasmids. Cells were fixed 24 h p.i. stained with NP-specific antibody and analyzed by CLSM. Lower panels show higher magnification of boxed area and white arrow indicates co-localization of nucleocapsid with mCherry-Tsg101 and IQGAP1-YFP in wild type infected cells whereas mutant nucleocapsids did not show any co-localization. (**C**) IQGAP1 depletion of infected cells. MARV-infected Huh-7 cells (MOI of 1) were transfected with IQGAP1-specific siRNA or control siRNA at 1 h p.i. Cells and culture supernatants were harvested at 48 h and 72 h p.i. Lysates and supernatants collected at 72 h p.i. were subjected to Western Blot analysis. (**D**) Virus titers in the supernatants of MARV infected cells transfected with IQGAP1-specific or control siRNA were determined by TCID_50_ titration, p-value (_*_, P≤0.05). (**E**) Phenotype of IQGAP1-knockdown in MARV-infected cells at 48 h p.i. Huh-7 cells grown on cover slips were infected with MARV_wt_ and treated with IQGAP1 specific or control siRNA and subjected to immunofluorescence analysis using NP-specific antibody. Grey boxes indicate marginal region of cells. Lower panels show higher magnification of boxed area, arrow indicates accumulation of nucleocapsids upon IQGAP1 knockdown at cell periphery marked with dashed line. Bars, 10 µm.

We have shown previously that transport of MARV nucleocapsids is dependent on the activity of the actin polymerization [26]. We were therefore looking for candidates that could link nucleocapsids via Tsg101 to the actin cytoskeleton. One likely candidate was the cellular factor IQ motif containing GTPase-activating-like protein (IQGAP1), which is involved in cytoskeletal dynamics and had been shown to interact with Tsg101 [8]. Control cells expressing fluorescent IQGAP1-YFP only displayed actin network-like distribution with some vesicular structures (Fig. 9A, upper panel right). The mCherry-Tsg101 showed a dot-like and vesicular appearance as observed with Tsg101-Venus1/2 (Fig. 9A, upper panel left). To analyze whether IQGAP1 was recruited by Tsg101 into NP-inclusions, we expressed IQGAP1-YFP and mCherry-Tsg101 together with NP_wt_ or NP_PSAPmut_ and VP40 and performed an immunofluorescence analysis. In cells expressing NP_wt_ together with IQGAP1-YFP and mCherry-Tsg101, the three proteins co-localized in NP-inclusions (Fig. 9A, white arrow middle panel). When mCherry-Tsg101 and IQGAP1-YFP were co-expressed with NP_PSAPmut_, neither mCherry-Tsg101 nor IQGAP1-YFP were localized to the inclusions. However, mCherry-Tsg101 and IQGAP1-YFP were detected co-localized outside of NP-inclusions in vesicular-like structures that were devoid of NP (Fig. 9A, white arrowhead lower panel). To confirm this observation, mCherry-Tsg101 and IQGAP1-YFP were co-transfected into rMARV_wt_ and rMARV_PSAPmut_ infected cells and analyzed by CLSM. In cells infected with rMARV_wt_ we found co-localization of mCherry-Tsg101 and IQGAP1-YFP in inclusions (Fig. 9B, white arrow in the merge panel) and with individual nucleocapsids (Fig. 9B, white arrow in the lower panel). In cells infected with the rMARV_PSAPmut_ we detected mCherry-Tsg101 and IQGAP1-YFP neither in NP-inclusions nor in association with individual nucleocapsids. (Fig. 9B, right row). We then asked whether siRNA-mediated down-regulation of IQGAP1 impaired MARV propagation. Western blot analysis showed IQGAP1 depletion in cells treated with IQGAP1-specific siRNA (Fig. 9C). Depletion of IQGAP1 resulted in a reduced release of MARV NP into the culture supernatant of the infected cells (Fig. 9C, right panel). It was then analyzed whether virus titers in the supernatant of the infected cells were also affected by the down regulation of IQGAP1. TCID50 analysis showed that virus titers dropped by 2–3 fold upon IQGAP1 depletion (Fig. 9D). In contrast, down regulation of IQGAP1 had no effect on the release of rMARV_PSAPmut_. This suggested that IQGAP1 acts through Tsg101, which is bound via the PSAP motif in NP to the nucleocapsid (Fig. 9B). The lower titer of rMARV_PSAPmut_ in comparison with rMARV_wt_ (Fig. 9D) corresponded to data presented in Fig. 2. In immunofluorescence analysis, MARV-infected IQGAP1-depleted cells were characterized by accumulations of nucleocapsids in the cell periphery similar as observed with Tsg101 depletion or rMARV_PSAPmut_ infection (Fig. 9E, see white arrows). Finally, we investigated by live cell microscopy whether IQGAP1 was co-transported with MARV nucleocapsids. We transfected MARV_VP30GFP_ -infected cells with IQGAP1-YFP and monitored the trajectories of individual nucleocapsids. We observed that IQGAP1 signals appeared like comet tails at the rear end of the rocketing nucleocapsids (movie S4, see along the white line). Altogether these results indicated that the PSAP motif of NP recruits Tsg101 and IQGAP1 to support efficient transport of nucleocapsids to the budding sites.

## Discussion

In this study, we explored the role of Tsg101 in the assembly process of MARV and showed that the PSAP-mediated interaction of NP with Tsg101 influences several intermediate steps of MARV assembly before viral fission. This phenotype is different from retroviruses, in which mutation of the PS/TAP motif in retroviral Gag domains results in defects of viral fission [2], [3]. Using electron tomography, it was possible to show that the number of intracellular nucleocapsids was enhanced in the rMARV_PSAPmut_-infected cells. In contrast, the number of completely enveloped particles was similar between the rMARV_PSAPmut_- and rMARV_wt_-infected cells. This suggested that the NP interaction with Tsg101 influences mainly pre-fission steps. The observed phenotype is clearly different from the “classical” late domain phenotype, which is characterized by the accumulation of completely enveloped viral buds connected to the cell by only a thin neck [37]–[41]. We previously showed that the ESCRT machinery influences the budding function of VP40, which recruits Nedd4-like ubiquitin ligases through a PPPY late domain motif [29]. Possibly, the recruitment of ESCRT by VP40 is sufficient to mediate the final fission step, whereas the interaction between NP and Tsg101 is necessary to support pre-fission steps, being mainly responsible for the transport of nucleocapsids into filopodia.

**Figure 10:**
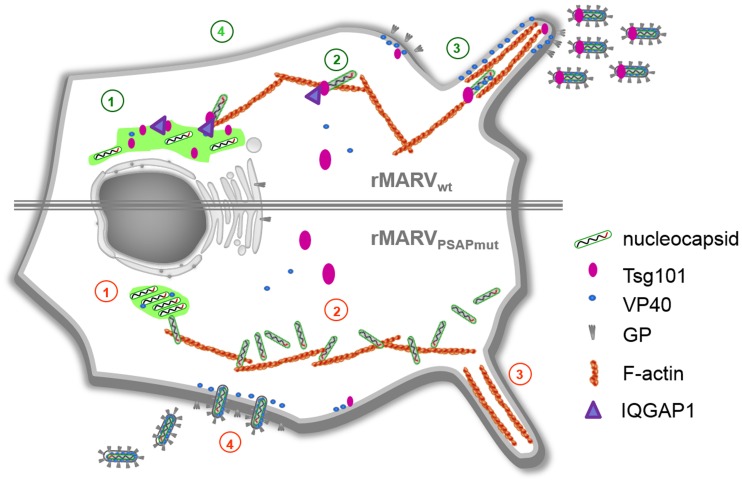
Schematic presentation of the rMARV_PSAPmut_ phenotype in comparison to rMARV_wt_. 1. rMARV_PSAPmut_ nucleocapsids accumulate in the inclusions which appear more dense and round. 2. The nucleocapsids accumulate in the cytosol and cell periphery. 3. rMARV_wt_ virus buds preferentially from filopodia. 4. rMARV_PSAPmut_ displays enhanced budding from the planar surface of cell. Numbers in red indicate the defective phenotype of MARV_PSAPmut_ and numbers in green show rMARV_wt_ infection.

Mutating the late domain motif PSAP in NP has previously been shown to impair its binding to Tsg101 and the release of MARV-specific VLPs [31]. Here, we demonstrate that rMARV_PSAPmut_, whose mutated NP is no longer able to bind Tsg101, displayed altered inclusion morphology and an increased accumulation of mature nucleocapsids inside inclusions and in the cytosol (Fig. 10, point 1). We hypothesized that this phenotype was the result of the missing interaction between NP and Tsg101, which reduced nucleocapsid transport efficiency. This idea was confirmed by live cell imaging data showing that Tsg101 was co-transported with wild type MARV nucleocapsids which was not observed in rMARV_PSAPmut_–infected cells. Recently published data showed that the intracellular actin-driven transport of MARV nucleocapsids proceeds in two steps: (i) nucleocapsids migrate from inclusions to the plasma membrane at a rate of 200 nm/s, and (ii) in the periphery, nucleocapsids are transported along the plasma membrane and inside filopodia at a rate of 100 nm/s [26]. Our data indicate that the speed of nucleocapsid transport from inclusions to the plasma membrane remains unaltered in rMARV_PSAPmut_-infected cells. However, the lengths of the trajectories of individual nucleocapsids were significantly shortened when the NP interaction with Tsg101 was disrupted. More importantly, the transport of nucleocapsids in the periphery of rMARV_PSAPmut_-infected cells was strongly impaired by the missing interaction between NP and Tsg101 (Fig. 10, point 2). This result was confirmed by EM studies. Using a whole-cell-mount method, we found that nucleocapsids in rMARVP_SAPmut_-infected cells were not efficiently recruited into filopodia, which represent the preferential budding sites for MARV (Fig. 8A and Fig. 10, point 3) [27], [28]. Recent studies indicated that the entry of nucleocapsids into filopodia depends on interaction with VP40, which takes place close to the plasma membrane [26], [42]. Because the interaction of NP_PSAPmut_ and VP40 is intact [31], the severely hampered movement of nucleocapsids in the periphery of rMARV_PSAPmut_-infected cells is likely caused by the transport defect of nucleocapsids.

The budding of viruses from filopodia is believed to contribute to more efficient cell-to-cell spread [43]–[45]. Indeed, the reduced delivery of nucleocapsids to filopodia may be responsible for the significantly impaired spread of rMARV_PSAPmut_ which mainly budded from the planar cell surface (Fig. 3A and Fig. 10, point 4).

Although the function of Tsg101 in supporting the formation of intraluminal vesicles at multivesicular bodies and during cytokinesis has been well studied, it is unclear how Tsg101 may function to support intracellular transport of nucleocapsids, as reported in this study. However, several publications suggest a potential link between Tsg101 and cytoskeleton activity. Tsg101 has been reported to interact with regulators of cytoskeleton dynamics, such as IQGAP and ROCK1 [8], and Tsg101 was essential for translocating the Src tyrosine kinase to cellular protrusions [9]. Together with small GTPases Rac1 and Cdc42, IQGAP1 controls dynamics of actin filament polymerization by different actions including actin bundling, barbed-end capping and binding actin branching as well as nucleating proteins [46], [47]. It has been shown that IQGAP1 supports egress of Moloney murine leukemia virus and classical swine fever virus [48], [49]. Recently it was published that IQGAP1-depletion of cells resulted in reduced release of EBOV VP40-induced VLPs [50]. We detected here recruitment of IQGAP1 into MARV inclusions and to individual nucleocapsids in dependence of the PSAP motif in the nucleoprotein. We also observed reduced virus release upon IQGAP1 depletion in infected cells. Based on these data in combination with the finding that the actin cytoskeleton is the dominant driver of MARV nucleocapsid transport [26], we hypothesize that Tsg101 mediates the interaction between viral nucleocapsids and IQGAP1 via the PSAP motif in NP. IQGAP1 then links Tsg101 to actin and simultaneously coordinates actin dynamics. Future studies are needed to test this hypothesis and elucidate how IQGAP1 and other cellular and/or viral factors mechanistically coordinate the intracellular transport of MARV nucleocapsids. This question is of particular interest because small molecule inhibitors interfering with the interaction of viral late domains with Tsg101 have recently been described as potential new antivirals [51], [52].

Mutation of the PSAP motif in NP severely impaired the incorporation of Tsg101 into viral particles but left the incorporation of multi-ubiquitinated Tsg101 intact. This phenomenon is currently not understood. The function of multi-ubiquitinated Tsg101 is still debated; for example, multi-ubiquitinated Tsg101 has been suggested to regulate endosomal trafficking by regulating the amount of active intracellular Tsg101 [12], [13]. Ubiquitinated Tsg101 has been reported to form insoluble complexes, which is in line with the observation that during siRNA down-regulation of Tsg101, the ubiquitinated form can be detected, whereas the non-ubiquitinated form is below the detection limit (Fig. 1B, [12]). Urata and colleagues reported in a previous study that Tsg101 can interact with VP40 in a PPPY-dependent manner and suggested later an indirect interaction with the involvement of an additional cellular factor [30], [53]. This interaction may be responsible for the recruitment of ubiquitinated Tsg101 into budding particles possibly via the arrestin-related proteins as suggested by Rauch and Martin-Serano [54].

Although the PSAP motif is a known binding motif for Tsg101 [55], , it cannot be completely ruled out that mutation of the PSAP motif in MARV NP inhibited the interaction with other cellular proteins that can bind similar motifs [57]. However, down-regulation of Tsg101 in the MARV-infected cells led to a phenotype similar to that observed in the rMARV_PSAPmut_-infected cells (Fig. 1E).

In summary, our results indicate that the efficient transport and envelopment of nucleocapsids depend on the PSAP motif in NP which recruits Tsg101 and in turn IQGAP1. The mutation of the PSAP motif in the recombinant rMARV_PSAPmut_ resulted in an intracellular accumulation of nucleocapsids caused by defective transport, which attenuated the efficient viral spread to neighboring cells.

## Materials and Methods

### Cell lines and viruses

Human embryonic kidney cells (HEK293; bought from the American Type Culture Collection (ATCC), Manassas, USA), human hepatoma cells (Huh-7; kindly provided by R. Bartenschlager, Heidelberg, Germany) and African green monkey kidney cells (Vero E6; from ATCC) were maintained in Dulbecco's modified Eagle medium (DMEM) supplemented with 10% fetal calf serum, L-glutamine and penicillin-streptomycin solution (Gibco, Karlsruhe, Germany). The Musoke strain of MARV was propagated on Vero E6 cells grown in DMEM supplemented with 3% fetal calf serum, L-glutamine and penicillin-streptomycin solution. Infections with MARV were performed under BSL-4 conditions at the Institute of Virology, Philipps University Marburg.

### Antibodies

Mouse monoclonal antibodies were used for the detection of MARV NP and VP40 in Western Blot. A goat anti-MARV serum was used for the detection of viral plaques in the immunoplaque assay. Mouse monoclonal antibodies anti-Tsg101 (GTX, San Antonio, USA), anti-IQGAP1 (Upstate, Biotechnology, Lake Placid, USA), anti-tubulin (Sigma-Aldrich, Deisenhofen, Germany), and rabbit anti-HA antibody (Rockland Immunochemicals, Gilbertsville, PA, USA) were used according to supplier's instructions. Biotinylated anti-Flag antibody M2 was purchased from Sigma (Sigma-Aldrich Biochemie GmbH, Hamburg, Germany). Secondary antibodies conjugated to horseradish peroxidase (HRP) were from Dako (Glostrup, Denmark). HRP-conjugated streptavidin was from GE Healthcare Bio-Science, Pittsburg, USA. Goat anti-mouse conjugated with Alexa Fluor 680- and IRDye 800-conjugated goat anti-rabbit secondary antibodies (Life Technologies GmbH, Darmstadt, Germany) were used for detection of proteins by the Li-Cor Odyssey imaging system according to supplier's instructions. For immunofluorescence analysis polyclonal guinea pig anti-NP sera was used. Secondary antibodies conjugated to fluorescein isothiocyanate (FITC) (Dianova, Hamburg, Germany), or Alexa 594 or Marina blue (Life Technologies GmbH, Darmstadt, Germany) were used for immunofluorescence analysis.

### Plasmids and mutagenesis

Cloning of full-length MARV cDNA clones was performed by amplifying non-coding 3′- and 5′-ends of the MARV genome from the MARV-specific minigenome [19]. Genomic viral RNA was used as RT-PCR template for coding and intragenic regions. The anti-genomic sequence of MARV Musoke (accession number: NC 001608) was cloned in three parts [Fragment 1 (FR1): T7 leader-NP-VP35-VP40-GP-; FR2: GP-VP30-VP24-L-; FR3: L-trailer-ribozyme)] flanked by unique restriction sites into three individual pBlueScript plasmids. Assembly of the full length plasmid containing the whole anti-genome of MARV Musoke was performed by standard ligation of the three DNA fragments into a minimal pBlueScript vector under the control of the T7 polymerase promoter [19]. To distinguish between recombinant (rMARV_wt_) and wild type MARV, silent mutations at position 6498 (C>T) and 7524 (A>G) were introduced resulting in the deletion of a KpnI restriction site and insertion of a SacII restriction site, respectively. Multi-site-directed mutagenesis was used to introduced mutations into the NP gene in FR1 resulting in amino acid substitution of the PSAP motif into AAAA and the PTAP motif into ATAA. Introduced mutations were confirmed by sequencing. In addition to the introduced mutations, two additional silent mutations at the nucleotide positions 7092 (G>A) in the GP gene and 15317 (G>A) in the L gene were detected in rMARV_wt_. For the construction of the plasmid pCAGGS-VP30-GFP coding for the VP30-GFP fusion protein, the GFP ORF was cloned in frame to the 3′ end of the VP30 gene using homolog recombination and primer-extension PCR. For dual-color live cell imaging a recombinant MARV coding the VP30RFP fusion protein was constructed (rMARV_VP30RFP_). The VP30RFP ORF was in addition inserted into an artificial AvrII restriction site between the VP35 and VP40 genes as described by Schudt et al. [26]. Sequencing of the viral RNA from rMARV_VP30RFP_ revealed no addition mutations to the indicated silent mutations in rMARV_wt_. Detailed cloning strategies as well as primer sequences are available upon request. Hemagglutinin-tagged ubiquitin (HA-Ub) was expressed from pCAGGS expression vector. The expression plasmid for IQGAP1 C-terminally fused to YFP (IQGAP1-YFP) was kindly provided by George S. Bloom [58]. Tsg101 N-terminally fused to mCherry (mCherry-Tsg101) was created by Quan Lu and provided by addgene (ID 38318) [59].

### Rescue of recombinant viruses

Vero and Huh-7 cells were mixed in a proportion of 1∶1 and grown in 6-well plates to 50% confluence. Transfection with support and full-length plasmids was performed as published earlier [60]. Culture supernatants were blind passaged on fresh sub-confluent Vero E6 cells 7 days post transfection (p.tr.) and cells monitored for cytopathic effect (CPE) development. Virus rescue was confirmed by Western Blot analysis of culture supernatants using MARV specific antibodies and by RNA isolation and sequencing. Sequencing of the viral RNA from rMARV_PSAPmut_ revealed in addition to the above indicated silent mutations in rMARV_wt_ additional mutations. An amino acid exchange V_396_ to L (nucleotide exchange at position 1289 G>T) in NP and at nucleotide position 2837 A to G mutation in the transcriptional stop codon of NP was found. Databank analyses revealed that aa 396 in NP of MARV strains Ravn and DRC99 is represented by Alanine indicating that this amino acid is not highly conserved. Our further studies revealed no changes in the proportion of NP to VP35 at protein level in lysates of rMARV_PSAPmut_- in comparison to rMARV_wt_–infected cells indicating that the mutation in the transcriptional stop codon of NP was not relevant within the scope of this study. In the L gene an insertion of three additional adenosines (nucleotide 12071-3) was detected which results in an insertion of a Lysine at the highly variable N-terminal region of L. Analyses of viral transcription and replication in cells infected with recombinant mutant and rMARV_wt_ revealed no differences. This result suggested that the insertion of the Lysine did not alter the ability of L to support viral transcription and replication. All experiments performed with recombinant mutant virus (rMARV_PSAPmut_) used recombinant wild type virus (rMARV_wt_) as control.

### Immunoplaque assay

Vero E6 cells were inoculated in 12-well plates with two-fold serial dilutions of rMARV_wt_ or rMARV_PSAPmut_ and 1 h post infection (p.i.) overlaid with 1.2% Avicel in MEM with 4% FCS. At day 4 p.i. the overlay was removed and cells were fixed with 4% paraformaldehyde (PFA) in DMEM. The next day the plates were completely covered with fresh 4% PFA and removed from the BSL-4 laboratory. Immunostaining was conducted 24 h later using goat anti-MARV specific sera and a secondary HRP-conjugated donkey anti-goat antibody. Immunoplaques were visualized after incubation with TrueBlue peroxidase substrate containing 0.03% hydrogen peroxide. The plates were dried and pictures of plaques captured utilizing a Nikon TS-100 microscope with a digital sight DS-SMC camera. Relative plaque areas were determined with Leica LAS AF software.

### Tissue culture infection dose 50 (TCID_50_) assay

Vero E6 cells were cultured in 96-well plates to 50% confluence and infected with 10-fold serial dilutions (eight replicates) of supernatants from infected cells. At 10 to 14 days p.i., when the CPE had stabilized, cells were analyzed by light microscopy. The TCID_50_/ml titers were calculated using the Spearman-Kärber method [61].

### Immunoprecipitation

For immunopreciputation of ubiquitinated Tsg101 a Flag-tagged Tsg101 was used as described earlier (Dolnik et al., 2010). Cells were transfected with Tsg101-Flag and HA-Ub expression plasmids and 48 h p.tr. lysed in Co-IP buffer (20 mM Tris-HCl, pH 7.5, 100 mM sodium chloride, 0.4% (w/v) deoxycholic acid, 1.0% Triton X-100, 0.5% (w/v) NP-40, 5 mM EDTA and 2% BSA) for 20 min at 4°C. Cell debris were removed by centrifugation at 14.000 rpm for 10 min. Lysates were incubated by with anti-HA agarose or anti-Flag agarose (Sigma-Aldrich) for 3 h at 4°C. Precipitates were washed 3 times with Co-IP buffer without Triton X-100, resuspended in samples buffer boiled and subjected to SDS-PAGE and Western blotting.

### Immunofluorescence analysis and confocal laser scanning microscopy

Immunofluorescence analysis was performed as described previously [31]. Images were taken either on Zeiss Axiophot upright fluorescence microscope using a Spot inside B/W QE digital camera (Visitron Systems, Puchheim, Germany) and VisiView image acquisition software, or on Leica SP5 confocal laser scanning microscope.

### Live cell imaging

For live cell imaging, Huh-7 cells were seeded into 35-mm μ-dishes (Ibidi, Munich) 24 h prior to infection. Cells were infected in Opti-MEM without phenol red (Life Technologies) for 1 h, then inoculum was removed and cells were transfected with plasmids encoding VP30-GFP or Venus1-Tsg101 and Venus2-Tsg101. Live cell time-lapse experiments were recorded with a Leica DMI6000B using a 63× oil objective equipped with a remote control device to operate the microscope from outside the BSL-4 facility. Pictures and movie sequences were processed with Leica LAS AF software.

### SDS-PAGE and western blot analysis

SDS-PAGE and Western Blot analysis were performed as described previously [62] and the intensity of bands was quantified using the Chemicon system and ImageLab software from Biorad. Dual protein detection was performed with Li-Cor Odyssey imaging system using fluorescent conjugated secondary antibodies as indicated in the antibodies section.

### Tsg101 and IQGAP1 knockdown in MARV-infected cells

Huh-7 cells were infected with MARV at a MOI of 1 and subsequently transfected with Tsg101-specific or IQGAP1-specific siRNA (Qiagen, Hs-TSG101-7, final concentration 20 nM; Hs-IQGAP1-3, final concentration 50 nM) or a control siRNA (Qiagen; control non-sil. siRNA) using Hiperfect transfection reagent. Second transfection was performed at 18 h p.i. and cells and supernatants were harvested at 48 h p.i. Tsg101 knockdown in cells was confirmed by Western Blot using Tsg101-specific antibody. Virus particles were pelleted from supernatants by ultracentrifugation and analyzed for Tsg101 incorporation by Western Blot. Protein levels were quantified using Image Lab software from Bio-Rad Laboratories. Virus titers in the supernatants were determined by TCID_50_ titration.

### Electron microscopy and tomography

Huh-7 and Vero E6 cells were grown on Thermanox cover slips, infected with rMARV_wt_ or rMARV_PSAPmut_ and fixed on the cover slips at 26 h p.i., then cells were processed for electron microscopy as described previously [18], [28]. Ultrathin (65 nm) and semi thick (300 nm) sections of the cell monolayers were cut nearly parallel to the plane of the cover slip with a Leica Ultracut UCT microtome (Leica Microsystems, Wetzlar, Germany). Thin sections were examined and imaged using a Zeiss EM10 TEM operated at 80 kV and a 1K×1K side mounted Gatan DualVision CCD camera. Electron tomography was carried out essentially as described elsewhere [28]. 10 nm gold fiducials were adsorbed to both surfaces of 300 nm thick sections on Formvar-coated grids and sections were post-stained with Reynold's lead citrate. Tilt series were recorded on a FEI Tecnai G2 F30 microscope, operated at 300 kV, using SerialEM software and a 4K×4K FEI Eagle CCD camera, at binned pixel sizes of 1.5 nm to 2.54 nm on the specimen level over a −60° to 60° tilt range (increment 1°) and at a nominal defocus of −1 µm [63]. Tomograms were reconstructed using the IMOD software package (version 4.1.4) [64]. 3D surface renderings were done with the AMIRA Visualisation Package (version 5.4.0, Visage Imaging, Berlin, Germany). Electron microscopy of whole mounted cells was performed as described previously [27].

### Morphometry of inclusions

Ultrathin sections of infected cells were stained with uranyl acetate and lead citrate and images were acquired on a Zeiss 109 electron microscope with 1K×1K side mounted CCD camera (Tröndle, Moorenweis, Germany). Stereological method described by Weibel et al. [65] was used to determine the volume density of nucleocapsid structures (V_Vnc_) inside viral inclusions. Briefly, the test system consisting of regular dots was randomly placed on electron micrograph of viral inclusion. Dots lying on preformed nucleocapsid (N_NC_) structures in inclusions (N_IC_) and dots lying outside these nucleocapsid structures but still inside inclusions (N_Cyto_) were counted separately. The ratios of N_NC_/N_IC_ and N_Cyto_/N_IC_ determined the relative volume (or volume density) of nucleocapsids and cytosol within inclusions.

### Quantification of nucleocapsid localization in tomograms

Nucleocapsid localisation outside viral inclusions was assessed and quantified in 3D tomograms from 300 nm thick sections of rMARV_wt_- and rMARV_PSAPmut_-infected Vero E6 and Huh-7 cells. The length of all nucleocapsid structures within tomograms was measured using the AMIRA Visualisation Package (Visage Imaging, Berlin, Germany), and only full-length nucleocapsids that were completely represented in the tomograms were included in the quantification [18], [28].

### Protein fragment complementation assay

Human Tsg101 was visualized in live cells using yellow fluorescent protein (YFP) based on protein fragment complementation assay. Briefly, fragments 1 (amino acids 1–158) and 2 (amino acids 159–239) of the YFP derivative Venus were generated by PCR using Venus1-GCN4-leucine zipper or Venus2-GCN4-leucine zipper constructs as templates (kindly provided by S.W. Michnick, University of Montreal, Canada) and subcloned into pCAGGS expression vector. Tsg101 was fused at its N terminus with either Venus fragment 1 or Venus fragment 2 resulting in the Venus-Tsg101 fusion chimeras designated Venus1-Tsg101 and Venus2-Tsg101. In addition, a 10-amino-acid flexible linker consisting of (Gly-Gly-Gly-Gly-Ser)×2 was inserted between the Venus fragments and the open reading frame of Tsg101.

### Statistical analyses

The presented data for each experiment represent the mean value and standard deviation of at least three independent experiments. The statistical significance was determined using Student's t test. Asterisks indicate statistically significant differences (*p<0.05, **p<0.01, ***p<0.001).

## Supporting Information

Figure S1 **Immunoprecipitation of ubiquitinated Tsg101 from infected cells.** Huh-7 cells were infected with MARV and subsequently transfected with plasmids encoding Tsg101-Flag and HA-Ub. At 48 h p.i., cells were lyzed and lysates subjected to immunoprecipitation with anti-Flag-agarose. Cell lysates and the obtained precipitates were separated by SDS-PAGE and analyzed by Western Blot using HA- and Flag-specific antibodies. The position of the ubiquitinated Tsg101 band is indicated by an arrow between 55 and 70 kDa. *: unspecific ubiquitinated cellular protein.
(TIF)

Figure S2 **Live cell imaging analysis of Tsg101-Venus1/2 and nucleocapsids motility in MARV-infected cells.** (**A**) Huh-7 cells were infected with rMARV_VP30RFP_ and subsequently transfected with plasmids encoding Venus1-Tsg101 and Venus2-Tsg101. Tsg101-Venus1/2 recruitment into MARV inclusions. At 28 h p.i., 273 pictures were acquired every 30 seconds. Shown are three exemplary pictures taken at indicated time points during the acquisition of the movie (Movie S2). Signal for VP30-RFP (nucleocapsids) is displayed in red and for Tsg101-Venus1/2 in green. (**B**) Co-transport between Tsg101-Venus1/2 and MARV nucleocapsids. Cells were infected and transfected as indicated in (A). At 46 h p.i., a sequence of 300 pictures was taken every 2.7 seconds (Movie S3). Panels show maximal projections of the VP30-RFP signals (red) and Tsg101-Venus1/2 signals (green) and and overlay of both signals (merge). Pictures were taken from movie S3 (Movie S3). Bars, 5 µm.
(TIF)

Movie S1 **Movement of nucleocapsids in MARV_PSAPmut_–infected cells is severely impaired in the cell periphery.** Huh-7 cells were infected with either rMARV_wt_ or rMARV_PSAPmut_ and transfected with VP30-GFP expression plasmid. At 28 h (rMARV_PSAPmut_) and 43 h p.i. (rMARV_wt_), cells were analyzed by time-lapse microscopy. Sequence shows signal for VP30-GFP labeled nucleocapsids. Acquisition: Sequence corresponds to 2 min; one frame was taken every second. Red circles: non-moving nucleocapsids.
(AVI)

Movie S2 **Tsg101-Venus1/2 is recruited into MARV inclusions.** Huh-7 cells were infected with rMARV_VP30RFP_ and subsequently transfected with Venus1-Tsg101 and Venus2-Tsg101 expression plasmids. At 28 h p.i., cells were analyzed by time-lapse microscopy. Sequence shows signal for VP30-RFP labeled nucleocapsids. Acquisition: Sequence corresponds to 136.5 min; one frame was taken every 30 seconds. Green: Tsg101-Venus1/2. Red: VP30-RFP. Bars, 10 µm.
(AVI)

Movie S3 **Co-transport of Tsg101-Venus1/2 with MARV nucleocapids.** Huh-7 cells were infected with rMARV_VP30RFP_ and subsequently transfected with Venus1-Tsg101 and Venus2-Tsg101 expression plasmids. At 46 h p.i., cells were analyzed by time-lapse microscopy. Sequence shows signal for VP30-RFP labeled nucleocapsids and Tsg101Venus1/2. Acquisition: Sequence corresponds to 840.7 seconds; one frame was taken every 2.475 seconds. Green: Tsg101-Venus1/2. Red: VP30-RFP. Bars, 10 µm.
(AVI)

Movie S4 **IQGAP1-YFP is recruited in the tail of rocketing MARV nucleocapsids.** Huh-7 cells were infected with rMARV_VP30RFP_ and subsequently transfected with IQGAP1-YFP expression plasmid. At 24 h p.i. cells were analyzed by time-laps microscopy. Sequence shows signals for VP30-RFP labeled nucleocapsids and for IQGAP1-YFP (see along the white line). Acquisition: Sequence corresponds to 115.6 seconds; one frame was taken every 2.34 seconds. Green: IQGAP1-YFP. Red: VP30-RFP. Bar, 10 µm.
(AVI)
