# Supplementary figures and images for: Interaction with Tsg101 Is Necessary for the Efficient Transport and Release of Nucleocapsids in Marburg Virus-Infected Cells
Source: PLoS Pathog. 2014 Oct 16;10(10):e1004463. doi: 10.1371/journal.ppat.1004463 (PMC4199773; doi:10.1371/journal.ppat.1004463)

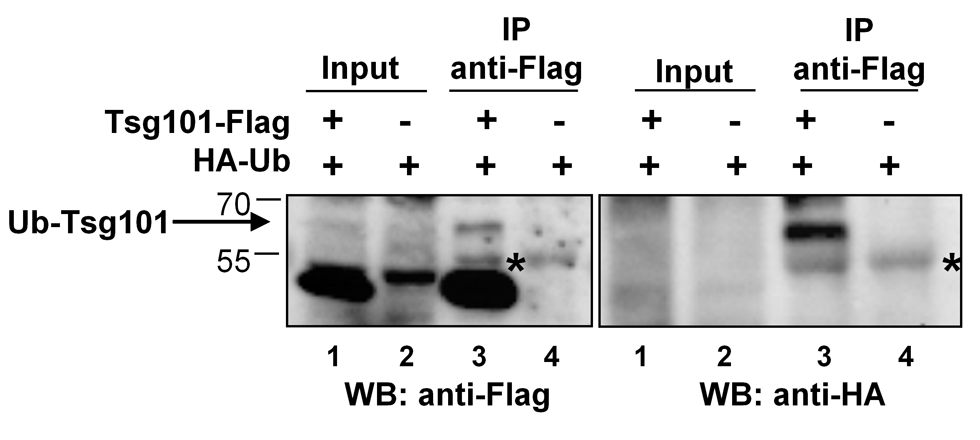

Supplement: Figure S1 — Immunoprecipitation of ubiquitinated Tsg101 from infected cells. Huh-7 cells were infected with MARV and subsequently transfected with plasmids encoding Tsg101-Flag and HA-Ub. At 48 h p.i., cells were lyzed and lysates subjected to immunoprecipitation with anti-Flag-agarose. Cell lysates and the obtained precipitates were separated by SDS-PAGE and analyzed by Western Blot using HA- and Flag-specific antibodies. The position of the ubiquitinated Tsg101 band is indicated by an arrow between 55 and 70 kDa. *: unspecific ubiquitinated cellular protein. (TIF) [file ppat.1004463.s001.tif]

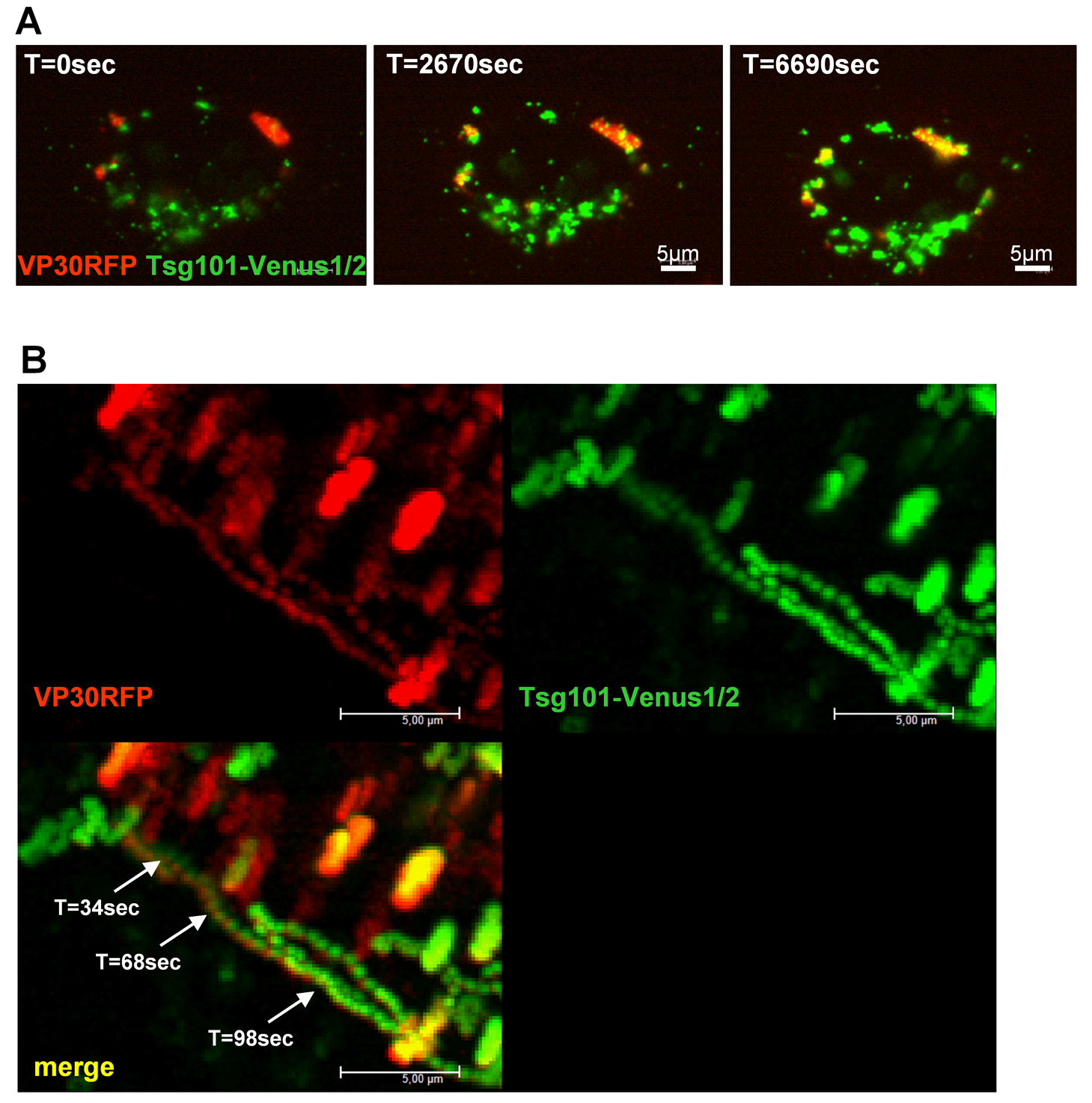

Supplement: Figure S2 — Live cell imaging analysis of Tsg101-Venus1/2 and nucleocapsids motility in MARV-infected cells. (A) Huh-7 cells were infected with rMARVVP30RFP and subsequently transfected with plasmids encoding Venus1-Tsg101 and Venus2-Tsg101. Tsg101-Venus1/2 recruitment into MARV inclusions. At 28 h p.i., 273 pictures were acquired every 30 seconds. Shown are three exemplary pictures taken at indicated time points during the acquisition of the movie (Movie S2). Signal for VP30-RFP (nucleocapsids) is displayed in red and for Tsg101-Venus1/2 in green. (B) Co-transport between Tsg101-Venus1/2 and MARV nucleocapsids. Cells were infected and transfected as indicated in (A). At 46 h p.i., a sequence of 300 pictures was taken every 2.7 seconds (Movie S3). Panels show maximal projections of the VP30-RFP signals (red) and Tsg101-Venus1/2 signals (green) and and overlay of both signals (merge). Pictures were taken from movie S3 (Movie S3). Bars, 5 µm. (TIF) [file ppat.1004463.s002.tif]
